# Correlating efficacy and desensitization with GluK2 ligand-binding domain movements

**DOI:** 10.1098/rsob.130051

**Published:** 2013-05

**Authors:** Naushaba Nayeem, Olga Mayans, Tim Green

**Affiliations:** 1 Department of Pharmacology, University of Liverpool, Liverpool L69 3GE, UK; 2 Institute of Integrative Biology, University of Liverpool, Liverpool L69 3GE, UK

**Keywords:** kainate receptor, desensitization, agonist-binding domain, dimer interface, anion binding

## Abstract

Gating of AMPA- and kainate-selective ionotropic glutamate receptors can be defined in terms of ligand affinity, efficacy and the rate and extent of desensitization. Crucial insights into all three elements have come from structural studies of the ligand-binding domain (LBD). In particular, binding-cleft closure is associated with efficacy, whereas dissociation of the dimer formed by neighbouring LBDs is linked with desensitization. We have explored these relationships in the kainate-selective subunit GluK2 by studying the effects of mutating two residues (K531 and R775) that form key contacts within the LBD dimer interface, but whose truncation unexpectedly attenuates desensitization. One mutation (K531A) also switches the relative efficacies of glutamate and kainate. LBD crystal structures incorporating these mutations revealed several conformational changes that together explain their phenotypes. K531 truncation results in new dimer contacts, consistent with slower desensitization and sideways movement in the ligand-binding cleft correlating with efficacy. The tested mutants also disrupted anion binding; no chloride was detected in the dimer-interface site, including in R775A where absence of chloride was the only structural change evident. From this, we propose that the charge balance in the GluK2 LBD dimer interface maintains a degree of instability, necessary for rapid and complete desensitization.

## 2. Introduction

**Figure 1.**
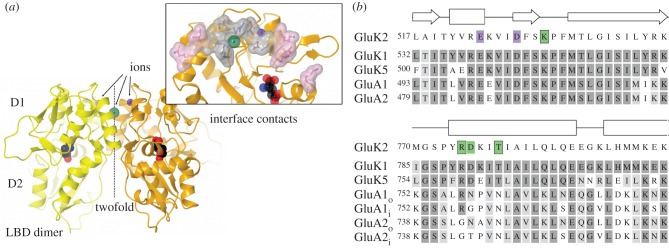
Apical interactions within GluK2 LBD dimer. (*a*) The GluK2 WT LBD dimer (PDB code 2xxr) [7] showing ligand (black), domains D1 and D2, the twofold axis and the Na^+^ and Cl^−^ ions (purple and green, respectively). Key D1:D1 interactions are shown inset on protomer B; pink surface from data in reference [8] and grey surface from this study. (*b*) Sequence alignment for kainate (GluK1, 2 and 5) and AMPA (GluA1 and 2; i and o indicate flip and flop splice variants). GluA2 is shown edited at the R/G site (743). Dark grey shading shows identity and light grey conserved. Secondary structure (from GluK2) is indicated by rectangles (α-helices) and arrows (β-strands). GluK2 residues involved in the cation and anion binding sites are highlighted in purple and green, respectively (mutated residues are boxed).

Both AMPA- and kainate-selective ionotropic glutamate receptors (iGluRs) desensitize rapidly (with time constants typically approx. 1–10 ms) and completely (by approx. 96–99.8%) in response to glutamate [1]. Desensitization of these ‘non-NMDA’ receptors involves rearrangement of a dimer formed by neighbouring ligand-binding domains (LBDs) [2,3]. The LBD is a clam-shell structure comprising an ‘upper’ (D1) and ‘lower’ (D2) lobe, with ligands bound between the two lobes, and the LBD dimer is formed by contacts across D1 lobes [4]. Desensitization is thought to occur on rearrangement of the dimer interface, breaking these D1:D1 contacts [3]. Consistent with this, macroscopic desensitization in both AMPA and kainate receptors (KARs) can be blocked by covalently linking (with disulphides) the LBD dimer [5,6]. Further, in both AMPA receptors and KARs, key inter-domain contacts are formed between residues at the edges of D1 (figure 1*a*, pink surface), and mutations to these edge sites can variously block [9] or attenuate [6,8,10] desensitization.

Alongside these structural similarities, there are also significant differences between the LBD dimers in AMPA receptors and KARs. Specifically, in KARs, additional contacts are formed at the dimer apex (figure 1*a*, grey surface), including a chloride ion bound on the twofold symmetry axis [11] and two sodium ions bound on either side [12]. Intriguingly, the residues forming the ion binding sites in kainate subunits are largely conserved in AMPA subunits (figure 1*b*). The only difference in primary sequence at the cation site is the substitution of the aliphatic residue capping the site in KARs by lysine in AMPA subunits. Of the two basic residues forming the anion site, the lysine is fully conserved but the arginine varies; it is subject to RNA editing in GluA2–4 (from arginine to glycine). In addition, an aspartate that forms a salt-bridge with the arginine in KARs varies in AMPA subunits depending on the splice variant (asparagine in ‘flop’, glycine or threonine in ‘flip’; figure 1*b*). These differences are apparently sufficient to occlude binding of ions to the LBD dimer interface in AMPA subunits [13].

To understand the functional implications of these differences, several groups have mutated apical residues both in AMPA [14] and in kainate subunits [11,15–17]. Most of these changes promote desensitization, although there are notable exceptions. Mutation of D776 to lysine in GluK2 blocks macroscopic desensitization [16], whereas mutation of GluK2 K531 to glycine, glutamate or alanine slows desensitization [15–17]. D776K results in a new, cross-dimer interaction [7], but there is currently no structural explanation for the K531 mutant phenotypes. We have addressed this question, characterizing three GluK2 apical mutants with attenuated desensitization (K531A, K531A-T779G and R775A) and identifying the conformational changes underlying their functional phenotypes.

## 3. Material and methods

### 3.1. Mutagenesis

Mutants were generated from a rat GluK2(Q) cDNA clone (previously known as GluR6). Residue numbering is for full-length subunits, except for GluA2 where it is for the mature polypeptide. Mutants were generated using the Quikchange protocol and *Pfu* Turbo or Ultra II polymerases (Stratagene, La Jolla, CA), as described previously [8]. All constructs were confirmed by sequencing.

### 3.2. Electrophysiology and data analysis

Electrophysiological recordings were carried out on outside-out patches pulled from transiently transfected HEK 293 cells 48–72 h post-transfection. Cell culture and recordings were carried out as described previously [8,18]. Rapid solution-exchange was achieved using a Burleigh LSS-3200 piezo-based system to drive movement of a theta perfusion tube relative to the patch. In recordings where chloride was replaced as the external anion, the CaCl_2_ and MgCl_2_ concentrations were reduced to 0.5 mM. Application times for glutamate (Glu; 10 mM) and kainate (KA; 1 mM) were selected based on the desensitization rates of the different mutants, and varied between 100 ms and 7 s (table 1). All data are presented as mean ± s.e.m.; unless otherwise stated, significant changes were assessed using one-way ANOVA followed by Dunnett's *post hoc* test to compare values with GluK2 wild-type (WT). The equilibrium constant for desensitization, *K*_eq_ (i.e. (desensitized)/(open)), was calculated from the per cent steady-state response (%SS) using the equation *K*_eq_ = (100−%SS)/%SS.

**Table 1.** Desensitization kinetics of GluK2 apical mutants. *τ*_des_, SS and *I*_KA/Glu_ are the desensitization time constant, steady-state current as a percentage of peak responses and relative kainate efficacy, respectively (mean ± s.e.m. for (*n*) determinations). All values were determined from outside-out patches pulled from transiently transfected HEK cells. Agonist applications varied as follows: GluK2 WT 100 ms (Glu and KA); K531A 0.4 s (Glu) and 4 s (KA); K531A-T779G 7 s (Glu and KA); R775A 0.4 s (Glu and KA). Double-exponential fits (*τ*_1_ and *τ*_2_) were used for responses that were poorly fitted by a single exponential. *τ*_1_ was the main component in all cases, and was used for statistical comparisons with GluK2 WT.

mutant	glu			KA	
	*τ*_des_ (ms)	SS (%)	*I*_KA/Glu_	*τ*_des_ (ms)	SS (%)
wild-type	3.7 ± 0.2 (*17*)	0.4 ± 0.1 (*10*)	0.53 ± 0.03 (*12*)	3.9 ± 0.3 (*11*)	1.64 ± 0.3 (*10*)
K531A	*τ*_1_ = 8.3 ± 1.0, *τ*_2_ = 125 ± 39 (*6*)	4.3 ± 0.6 (*7*)	1.22 ± 0.13*** (*5*)	*τ*_1_ = 92 ± 30*, *τ*_2_ = 925 ± 190 (*4*)	18.0 ± 1.4*** (*4*)
K531A-T779G	*τ*_1_ = 190 ± 20***, *τ*_2_ = 1460 ± 150 (*15*)	13.2 ± 1.6*** (*15*)	0.73 ± 0.03* (*7*)	*τ*_1_ = 220 ± 40***, *τ*_2_ = 2960 ± 900 (*6*)	67.0 ± 1.1*** (*7*)
R775A	*τ*_1_ = 10.1 ± 0.7, *τ*_2_ = 74 ± 16 (*9*)	4.0 ± 0.5 (*9*)	0.59 ± 0.03 (*7*)	*τ*_1_ = 16.4 ± 2.1, *τ*_2_ = 230 ± 130 (*7*)	20.6 ± 1.9*** (*7*)

*p < 0.05, ***p < 0.001

### 3.3. X-ray crystallography

GluK2 LBD constructs were generated, purified and crystallized as described previously [7]. Auto-induction (26°C for 20 h) was used for all constructs with the exception of GluK2 K531A-T779G, where expression was induced with isopropylthio-β-galactopyranoside (1 mM, 24°C for 4 h). Protein (in 25 mM HEPES pH 7.5, 150 mM NaCl, 5% glycerol with either 5 mM glutamate or 1 mM KA) was mixed 1 : 1 with reservoir (containing 19–27% PEG 4000, 0–9% propan-2-ol, 80 mM sodium acetate) for crystallization by hanging drop. All complexes were grown directly with the respective ligand, with the exception of K531A-T779G:KA, which was grown by soaking a glutamate-containing crystal in 1 mM KA. Diffraction data were collected at 100 K at Diamond beamlines I02 and I03 (Didcot, UK; ADSC CCD detectors) and at BESSY-II beamline MX 14-2 (Berlin, Germany; MAR CCD detector) as follows: K531A:Glu (I02), K531A:KA (I02), K531A-T779G:Glu (MX 14-2), K531A-T779G:KA (I03), R775A:Glu (MX 14-2) and R775A:KA (I03). Anomalous datasets were collected at *λ* = 1.5498 Å (K531A-T779G:Glu at I03 and R775A:Glu at I02; others as above), with the exception of K531A:Glu, where the anomalous signal in the standard dataset was used. Data processing and molecular replacement were carried out using xds/xscale [19] and phaser [20], respectively. GluK2:Glu (Protein Data Bank (PDB) accession code 2xxr) and GluK2:KA (2xxt) LBD structures [7] were used as initial models, with all mutated sites truncated to glycine. In addition, both R775 and D776 were truncated to alanine for any structures containing the K531A mutation. Refinement was carried out using either refmac5 (for K531A:KA) [21] or phenix.refine [22]. Programs from the CCP4 suite were used for various data manipulations [23], and coot [24] was used to visualize and manipulate models. Where used, TLS groups were identified using the TLSMD server [25].

Density for the main chain was continuous, with the exception of some residues within loops 1 and 2 in K531A:KA, K531A-T779G:Glu and K531A-T779G:KA. These were omitted from the final model. In the K531A-T779G:KA structure, the ligand density indicated mixed occupancy of the protomer D binding pocket by KA and Glu (occupancies refined to 61% and 39%, respectively). The C:D dimer was therefore omitted from analyses of conformational changes. Inter-dimer movements were analysed using dyndom [26] as described previously [7]. avepdb, lsqman and moleman2 programs from the USF suite (http://xray.bmc.uu.se/usf) were used to calculate averaged coordinates, determine per-residue r.m.s. differences, and calculate centres of mass, respectively. Structure figures were generated with either ccp4mg or pymol (see figures 4*e* and 5*a*,*b*). Electrostatic potentials were calculated using default settings in the pymol plug-in to the apbs program [27], with charges calculated by pdb2pqr [28] using the parse forcefield. Model coordinates and diffraction data have been deposited at the PDB for the GluK2 K531A:Glu (4bdl), K531A:KA (4bdm), K531A-T779G:Glu (4bdn), K531A-T779G:KA (4bdo), R775A:Glu (4bdq) and R775A:KA (4bdr) structures.

## 4. Results

### 4.1. Mutations to basic residues at GluK2 ligand-binding domain dimer apex attenuate desensitization

The anion and cation binding sites formed in the interface between kainate subunit LBDs play an essential role in receptor function [17,29]. Unsurprisingly, a wide range of mutations have been made to side chains in the vicinity, the majority of which result in smaller responses and/or faster desensitization kinetics [11,12,15,30]. This is consistent with the proposed role of ion binding in stabilizing the dimer interface [29]. An exception to this pattern is K531, which interacts with the chloride ion bound at the dimer twofold axis in GluK2. It also forms an inter-subunit salt-bridge with E524, which in turn interacts with the cation. Mutation of K531 to either glycine (K531G) [15], glutamate (K531E) [16] or alanine (K531A) [17] unexpectedly slows the rate of desensitization. In all three cases, these mutations would be expected to disrupt inter-subunit interactions, including the binding of chloride. By contrast, mutations to the equivalent site to GluK2 K531 in the AMPA-selective subunit GluA2 (K493A and K493M) resulted in faster desensitization, as would be expected [14].

**Figure 2.**
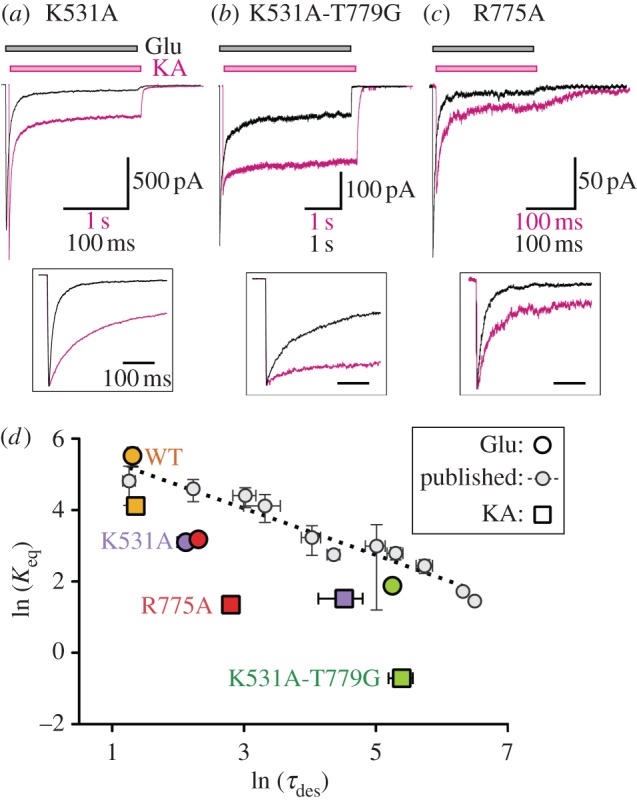
Functional effects of K531A, K531A-T779G and R775A mutations on GluK2 responses to Glu and KA. (*a*) Responses to applications of 10 mM Glu (400 ms; black trace) and 1 mM KA (4 s; magenta trace), recorded from outside-out patches pulled from HEK cells transfected with K531A. The traces are shown on a different time-base and horizontally offset to allow easier comparison of relative efficacy and steady-state responses. The inset shows the same traces on an equal time-base and with normalized peak heights, to allow comparison of desensitization rates. (*b*,*c*) Traces recorded in response to Glu and KA applications (7 s in (*b*); 0.4 s in (*c*)) from cells transfected with K531A-T779G and R775A respectively (displayed as in (*a*)). (*d*) Graph comparing the steady-state equilibrium constant (*K*_eq_) with the first-order desensitization time constant (*τ*_1_). Values are plotted as the natural logarithm (mean ± s.e.m.), making them proportional to free-energy of desensitization (*ΔG*_des_) and activation energy (*E*_a_ or *ΔG*^++^), respectively. The dotted line is the linear regression (*r*^2^ = 0.94) through data points (small light grey circles; ‘published’) calculated from steady-state and desensitization-rate data for the ten GluK2 mutants plus WT described by Chaudhry *et al.* [10].

In order to explore this further, we characterized the functional effects of three GluK2 mutants with truncated residues in this region: K531A, K531A-T779G and R775A. The first of these has been reported to affect both desensitization kinetics and agonist efficacy [17]. Consistent with this, we found that the K531A mutation results in slowly desensitizing responses with significant steady-state currents when either Glu or KA were applied to outside-out patches pulled from HEK cells expressing the mutant (figure 2*a* and table 1). Again, as previously described, the efficacy of KA is higher than Glu at this mutant, although whether this results from an increase in KA efficacy or a reduction in Glu efficacy cannot be determined from macroscopic responses. The second mutant incorporates a further mutation, T779G, which in isolation selectively slows desensitization of responses to KA [15]. When combined with K531A, this mutation causes further attenuation of desensitization in responses to both Glu and KA (figure 2*b* and table 1). Steady-state responses, in particular, were increased relative to K531A alone. In the third mutant, R775A, the second basic residue forming the anion binding site was also truncated to alanine. In previous studies, the conservative R775K mutation was found to increase desensitization rates [11,30]. Truncation of the side chain had the opposite effect, attenuating both the rate and extent of desensitization (figure 2*c* and table 1). Responses of GluK2 R775A to Glu and KA exhibited slower rates of desensitization compared with GluK2 WT, along with steady-state responses of similar magnitude to those observed in GluK2 K531A (table 1). Unlike K531A, neither this mutant nor K531A-T779G affected the efficacy of KA relative to Glu (table 1).

These changes in receptor responses can be considered in terms of energy states. One correlation that has often been highlighted is between the desensitization rate constant (*k*_des_) and LBD dimer stability. Chaudhry *et al.* [10] observed a linear correlation (*r*^2^ = 0.86) between GluK2 dimer stability (plotted as the *ΔG* of dimer dissociation) and the relative desensitization rate (plotted as −RTln(*k*_des_/*k*_WT_)). If, as is commonly assumed, peak and steady-state responses arise from the same open-state, then we would expect a similar relationship between the desensitization rate and the steady-state current (%SS, from which *K*_eq_ can be calculated). In energy terms, ln(*K*_eq_) should therefore be proportional to *ΔG*_des_. Similarly, the desensitization rate should be related to the activation barrier (*ΔG*^++^) between open and closed/desensitized states. In this case, the desensitization time constant (*τ*_des_) should be proportional to exp(*ΔG*^++^), with a higher activation energy slowing desensitization. We therefore plotted ln(*K*_eq_) against ln(*τ*_des_) to explore the effects of mutants on the thermodynamics and kinetics of desensitization (figure 2*d*). For the mutants described by Chaudhry *et al.* [10], this comparison actually results in a stronger correlation (*r*^2^ = 0.94). While correlation does not prove causation, this link is still interesting in terms of understanding KAR gating. The same inverse relationship is present, albeit weaker and with a different slope, for our apical mutants (*r*^2^ = 0.72; figure 2*d*). In general, the change in *K*_eq_ (i.e. steady state) in the apical mutants is larger than that seen in edge mutants with comparable effects on the desensitization rate. In other words, the apical mutations are affecting *ΔG*_des_ to a greater extent than *ΔG*^++^. The basis for this difference is considered further in §5.

### 4.2. Localized structural effects of mutations at ligand-binding domain dimer apex

**Figure 3.**
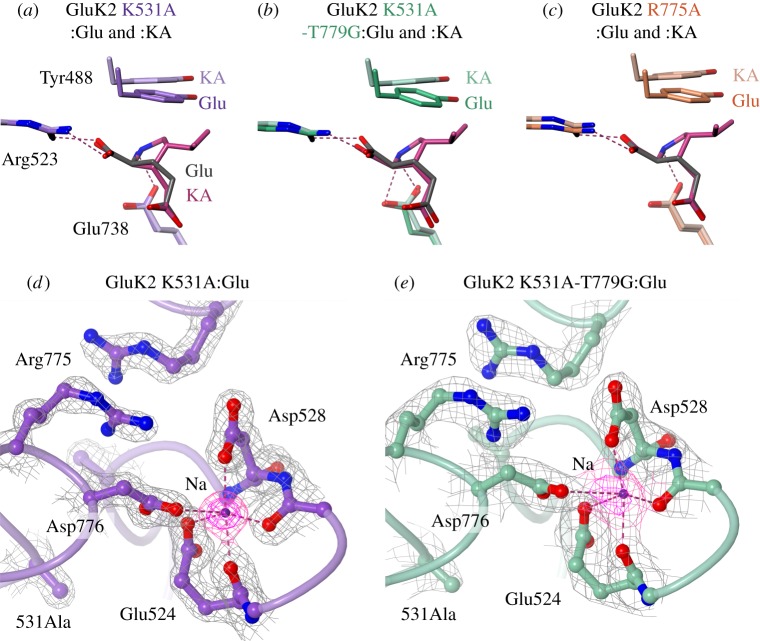
Ligand-binding cleft and dimer-interface interactions in GluK2 mutant LBD structures. (*a*) Simplified view of the ligand-binding sites in K531A:Glu (purple) and K531A:KA (light purple). Glu and KA are shown grey and magenta, respectively. Side chains directly interacting with ligand (R523 and E738; magenta dashes indicate H bonds) are shown, along with Y488, which is shifted upwards in the KA complex. (*b*,*c*) Equivalent views for K531A-T779G:Glu and :KA (green and pale green) and R775A:Glu and :KA (orange and pale orange). (*d*) Region around protomer B cation binding site in K531A:Glu, viewed from ‘above’ the LBD dimer (approx. 35° from the twofold axis). Key side chains and main chain atoms are shown as ball-and-stick. Contacts with the sodium ion (purple sphere) are indicated by magenta dashes. Electron density, (2*F*_obs_ − *F*_calc_)*α*_calc_, is shown around the displayed residues (grey mesh; contoured at 1.5 *σ*), and sodium (magenta mesh; contoured at 1.5 *σ* and 3.0 *σ*). (*e*) View of K531A-T779G:Glu, displayed as in (*d*).

Existing crystal structures provide no indication of how these mutations might attenuate desensitization. We therefore determined GluK2 LBD structures for the three mutants K531A, K531A-T779G and R775A in complex with either Glu or KA (table 2). The mutations had no effect on the overall bi-lobate fold observed in wild-type GluK2 LBD structures [31,32]. All three mutant LBDs associated as dimers, with either one (I222 form) or two (P2_1_2_1_2_1_ form) dimers in the asymmetric unit (table 2). The binding mode of the two ligands was also unaffected by the various mutations (figure 3*a–c*). Specifically, contacts between the ligand, the polypeptide and (where visible) the waters in the binding pocket matched those observed previously in GluK2 [7,31]. Comparing the Glu and KA complexes, the only significant difference was the position of Y488, which moves approximately 1 Å to accommodate the KA methyl group (figure 3*a*–*c*), again consistent with earlier GluK2 LBD structures.

**Table 2.** Data collection and refinement statistics for GluK2 mutants.

dataset	K531A		K531A-T779G		R775A	
ligand (space group)	:Glu (I222)	:KA (P2_1_2_1_2_1_)	:Glu (P2_1_2_1_2_1_)	:KA (P2_1_2_1_2_1_)	:Glu (I222)	:KA (I222)
wavelength (Å)	0.9795	0.9795	0.9184	0.9500	0.9184	0.9700
unit cell (a, b, c) (Å)	97.6, 106.6, 113.9	85.7, 101.0, 127.0	85.7, 100.1, 126.2	85.8, 99.8, 125.0	95.8, 105.6, 114.6	95.5, 105.3, 112.2
resolution (Å)^a^	33.9–1.75 (1.78–1.75)	46.9–3.40 (3.49-3.40)	32.6–2.50 (2.56–2.50)	40.8–2.55 (2.62–2.55)	33.6–1.90 (1.94–1.90)	19.8–1.65 (1.67–1.65)
unique reflections^a^	59 953 (2950)	14 888 (1151)	38 142 (2792)	35 532 (2592)	45 858 (2721)	66 578 (2378)
mean redundancy^a^	4.5 (4.5)	3.4 (3.4)	4.4 (4.4)	3.4 (3.5)	3.7 (3.6)	4.4 (4.1)
completeness (%)^a^	99.8 (99.9)	94.7 (95.1)	99.6 (99.8)	99.5 (99.8)	99.6 (99.2)	97.7 (99.8)
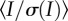 ^a^	15.5 (2.2)	6.6 (2.5)	12.4 (2.6)	12.1 (2.1)	14.6 (1.9)	21.2 (2.2)
*R*_symm_ 〈*I*〉^a^	6.6 (77.1)	26.6 (63.9)	11.3 (62.5)	8.8 (70.3)	7.0 (82.0)	3.5 (67.9)
*R*_work_/*R*_free_ (%)^b^	16.9/20.2	23.9/28.9	19.8/24.8	19.1/24.1	16.6/21.3	16.4/19.7
protomers (NCS dimers)	2 (1)	4 (2)	4 (2)	4 (2)	2 (1)	2 (1)
no. protein atoms	3972	7897	7902	7749	3975	4017
no. ligand atoms	20	60	40	60^c^	20	30
no. waters	503	—	337	154	461	489
bond/angle (°)/r.m.s.ds (Å)	0.006/1.02	0.014/1.48	0.006/0.72	0.008/0.89	0.007/0.99	0.006/1.08

^a^Values in parentheses represent the highest resolution shell.

^b^5.0% of reflections were excluded for calculation of *R*_free._

^c^In protomer D, ligand was modelled as both KA (occupancy 61%) and Glu (39%).

**Figure 4.**
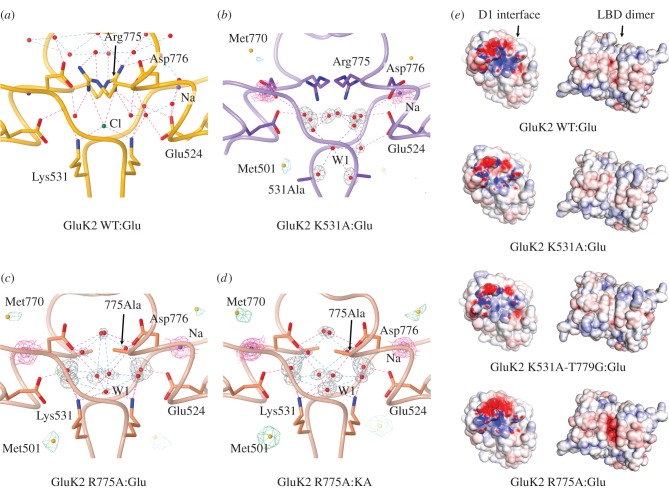
The anion binding pocket in GluK2 K531A and R775A mutants. (*a*) Ion binding sites in WT:Glu LBD structure (PDB code 2xxr), viewed perpendicular to the dimer twofold. Na^+^ and Cl^−^ ions (purple and green, respectively) are shown, along with key side chains and interacting waters. (*b*) Equivalent view for K531A:Glu. Electron density, (2*F*_obs −_ *F*_calc_)*α*_calc_, is shown around the sodium ions (magenta mesh, 1.5 *σ* and 4.0 *σ*) and waters (grey mesh, 1.5 *σ*) only. Anomalous difference-Fourier electron density (green mesh, 3.5 *σ*) is also shown. Clear anomalous-difference peaks are observed at buried methionine sulphur atoms (M501, yellow spheres), with weaker peaks at surface sulphurs (M770). W1 indicates the modelled water closest to the chloride site, but no significant (greater than 2.5 *σ*) anomalous difference peaks were associated with this or any other solvent molecule in the vicinity. (*c*,*d*) Equivalent views for the R775A:Glu and R775A:KA complexes, with anomalous difference-Fourier electron density contoured at 4.0 *σ*. Density modelled as solvents (W1) was present near the usual chloride ion binding site, but, again, no significant anomalous difference peaks were present. (*e*) Electrostatic surface potential calculated for the dimeric LBD in the absence of ions. Images on the left show the D1 interface on protomer A (red = −10 *k*_b_*T*/*e*_c_; blue = +10 *k*_b_*T*/*e*_c_), whereas images on the right show the intact LBD dimer viewed from ‘above’ (−8 to +8 *k*_b_*T*/*e*_c_). Distinct binding sites for sodium (red; both views) and chloride (blue; interface view) are visible in the GluK2-WT:Glu complex, as described previously [11,12]. The sodium binding site is still present in the mutants, but the chloride binding site is either poorly defined (K531A-containing mutants), or partly masked by more negative surface electrostatics (R775A mutant).

While the LBD fold and ligand binding were unaffected, there were significant changes in the immediate surroundings of the mutated residues. Two main effects were observed: re-arrangement of charged side chains neighbouring the truncated residues, and a reduction/loss of chloride binding. Changes in side-chain conformation were evident only in the GluK2 K531A and K531A-T779G LBD structures. In these mutants, both the R775 and D776 side chains adopt different conformations from GluK2 WT (figure 3*d,e*). These residues usually form a salt-bridge across the LBD dimer twofold axis, presumed to stabilize the complex (figure 4*a*). In the GluK2 K531A and K531A-T779G LBD structures, in contrast, opposing R775 side chains adopt an anti-parallel interaction across the dimer twofold, whereas D776 forms an inter-protomer contact with the neighbouring sodium ion (figure 3*d*,*e*). Sodium ions bind to proteins with a preferred octahedral geometry, and an average Na–O distance of 2.3–2.4 Å [33]. The D776–sodium contact maintains this geometry, although with a slightly longer contact distance of 2.6–2.8 Å in K531A:Glu and 2.9–3.3 Å in K531A-T779G:Glu and :KA (unrestrained during refinement). Collectively, these changes are reminiscent of those in the non-desensitizing GluK2 mutant D776K. That mutation results in an anti-parallel interaction between R775 guanidinium groups and new inter-protomer contacts (between the introduced lysine and the cation binding pocket) are the key features increasing dimer stability [7]. These analogous changes in K531A and K531A-T779G should therefore also increase LBD dimer stability.

### 4.3. Anion binding and anion-mediated functional effects

This raises the question why, if these contacts increase stability, they are not formed in GluK2 WT. The answer is probably the altered charge balance in this region, including the apparent loss of chloride binding when K531 is truncated. In GluK2, a chloride ion normally sits on the dimer twofold axis, in an electro-positive binding pocket mid-way between the basic residues K531 and R775 [29] (figure 4*a*). By contrast, in the K531A and K531A-T779G structures in complex with Glu, electron density in this region was either weak or absent. Anomalous difference-Fourier electron density maps showed no peaks consistent with chloride binding for either K531A:Glu (figure 4*b*) or K531A-T779G:Glu (data not shown). In both cases, significant anomalous difference peaks were visible for sulphur atoms in the vicinity of the dimer interface, but not in the solvent-filled void between residues 531 and 775.

From the structural data, it therefore appears that truncation of K531, and the resulting loss of two positive charges from the dimer interface, disrupts chloride binding. In contrast to what might be predicted, however, the lack of well-defined chloride binding is associated with attenuated desensitization in these mutants. This implies that the dimer is more stable in K531A and K531A-T779G than in GluK2 WT. In this respect, the GluK2 R775A structures were even more interesting. The interface-anion also appeared to be absent from the R775A:Glu and :KA LBD structures. While electron density was visible above K531, anomalous difference-Fourier electron density maps indicated this was solvent rather than chloride (figure 4*c*,*d*). Anomalous density consistent with a single bound chloride ion was observed in the R775A:KA structure, but this was at a separate site. In some respects, this is analogous to the situation in AMPA receptors containing glycine at the R/G site, with the important distinction that sodium ions are still bound to GluK2 R775A.

To assess the changes in the ion binding pockets, we calculated electrostatic potentials for the three mutant LBD dimers in complex with Glu, and compared these with the equivalent GluK2 WT complex (figure 4*e*). The internal D1 interface showed significant changes in the overall charge distribution (figure 4*e*, left images). While distinct regions of positive and negative potential corresponding to the sodium and chloride binding pockets are visible in GluK2 WT, this is not the case with the three mutants. The cation binding site appears generally unchanged, but the chloride binding site is reduced in either extent (R775A:Glu) or magnitude of the positive potential (K531A-containing mutants). In addition, for R775A:Glu, the region of negative potential extended significantly beyond the cation binding pocket, potentially masking the anion binding site from the solvent. This region of negative potential is visible on the surface of the LBD dimer (figure 4*e*, right images). Whereas the sodium binding sites can be seen as two discrete regions of negative potential in GluK2 WT [12], the negative potential extends across the dimer interface in R775A. In summary, the observed electrostatic potentials are fully consistent with the loss of chloride binding from the three mutants. The surprising point remains that this loss of chloride is the *only* change apparent in GluK2 R775A, a mutant with attenuated desensitization.

Exchange of chloride with other anions (i.e. F^−^, Br^−^, I^−^ or 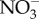) has two effects on macroscopic GluK2 responses: desensitization is faster, and peak responses are smaller [11,30]. We tested GluK2 K531A-T779G responses in NaCl and NaI to determine whether the absence of chloride binding in our structures was accompanied by changes in anion sensitivity. Surprisingly, responses were still affected by the identity of the external anion. Specifically, both the rate and extent of desensitization were altered when responses to Glu were measured in 150 mM NaI (*τ*_1_ = 44 ± 4 ms, *τ*_2_ = 690 ± 100 ms, %SS = 3.6 ± 0.3%; *n* = 3) compared with NaCl (*τ*_1_ = 150 ± 34 ms, *τ*_2_ = 1320 ± 320 ms, %SS = 9.6 ± 1.3%; *n* = 4). The differences in *τ*_1_ and %SS (but not *τ*_2_) were significant (*p* < 0.05; two-tailed *t*-test). It was less clear if there was a change in amplitude, with only a small, non-significant reduction in relative responses (0.90 ± 0.06). The structural data show that chloride binding to the dimer-interface site is significantly attenuated. Functional effects must therefore result from either residual binding of anions to the site, or binding of anions to additional sites in the receptor. Further electrophysiological investigation will be required to distinguish between those possibilities.

### 4.4. Changes to GluK2 ligand-binding domain conformation in apical mutants

The new inter-dimer contacts that we observed in the K531A and K531A-T779G LBD structures are consistent with attenuation of desensitization through greater dimer stability. They do not, however, fully explain the mutant phenotypes. Specifically, the K531A mutation affects relative efficacy when introduced on its own, but not in combination with T779G, whereas the double mutant has significantly larger steady-state responses. We therefore looked for differences in the overall conformation of the LBD dimer. The LBD can undergo two rigid-body movements: shifts at the D1:D1 dimer interface, and movement of D2 relative to D1 within a single protomer (e.g. cleft closure). The former has been implicated in desensitization [3,7], and the latter in gating and ligand efficacy [31,34]. Given the location of these mutations in the dimer interface, we first looked at the conformation of the D1:D1 interface. We had found in a previous study that this was affected by both mutation (i.e. D776K) and the nature of the bound ligand [7].

**Figure 5.**
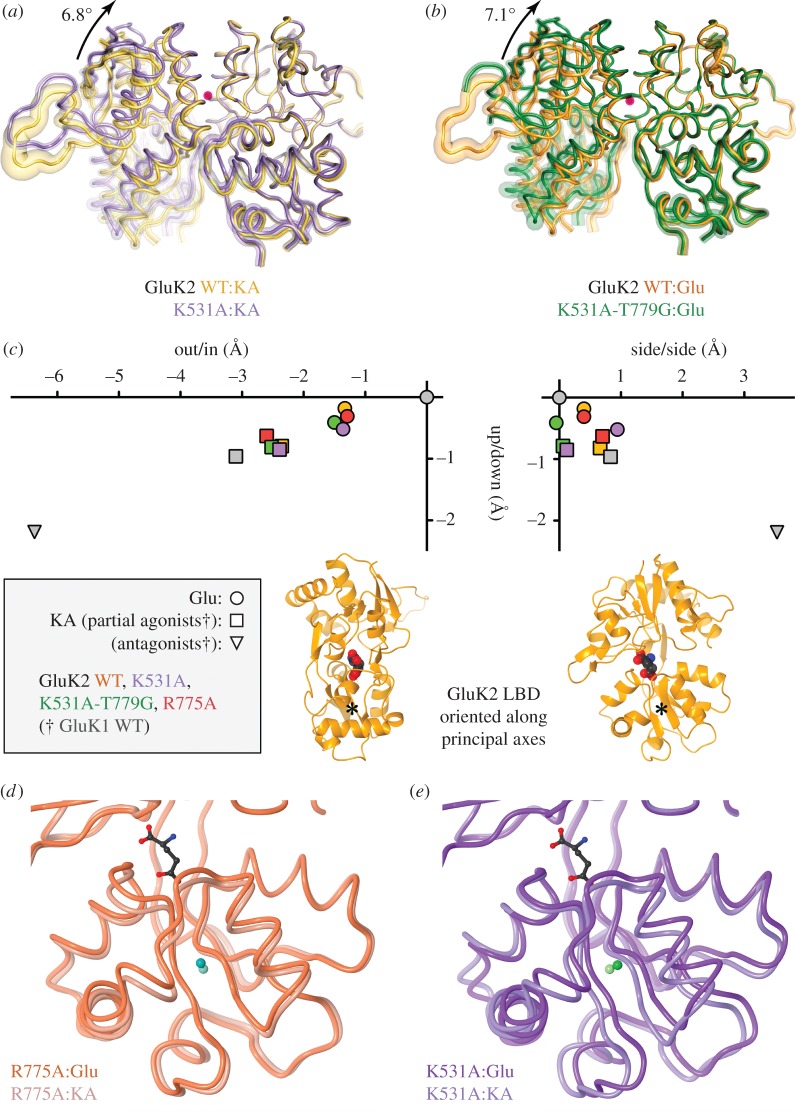
Domain movements in apical GluK2 mutants. (*a*) Comparison of D1:D1 dimer conformation in WT:KA and K531A:KA structures. Cartoons show averaged Cα coordinates determined following alignment to the D1 domain of GluK2 WT protomer A. Variability is indicated by tube diameter (transparent overlay where >0.4 Å; see §3). Structures are viewed down the axis for D1:D1 movement (magenta dot; magnitude of rotation indicated). (*b*) Equivalent view comparing WT:Glu and K531A-T779G:Glu. (*c*) Movement of D2 domain centres of mass along principal axes showing correlations with ligand efficacy. The left graph shows ‘cleft closure’ (i.e. up/down and in/out movement) and the right graph ‘twist’ (up/down and side/side). The orange cartoons illustrate the protomer orientation for the graphs above (asterisk marks D2 centre of mass). Points (see boxed key) are plotted relative to the 3fuz GluK1 WT:Glu complex (grey circle). For GluK1 structures, the points are *average* movements from selected structures of partial agonists (AHCP, 2wky; KA, 3c32; Dom, 2pbw) and antagonists (ATPO, 1vso; UBP310, 2ojt; a thiophene derivative, 3s2v). Efficacy is correlated with both cleft closure (left graph) and sideways shifts (right graph). (*d*) Cartoon of R775A:Glu and :KA LBD (averaged Cα coordinates), aligned on the D1 domain. The D2 domain of the Glu complex is shifted up and to the right compared with the KA complex (see centres of mass, indicated by dark and light cyan spheres). (*e*) Equivalent view comparing K531A:Glu and :KA, showing reversal of sideways shift in this mutant.

Comparing the interface conformations in the three apical mutants with those of the equivalent GluK2 WT complexes, movements were observed with K531A and K531A-T779G, but not with R775A (table 3). For K531A:Glu and K531A:KA complexes, there were interface shifts of 5.1° (table 3) and 6.8° (figure 5*a*) compared with their WT counterparts. Similarly, in the K531A-T779G:Glu and :KA complexes, the interface was rotated by 7.1° (figure 5*b*) and 6.6° (table 3) relative to the GluK2 WT equivalents. The axes of these rotational shifts were all essentially perpendicular to the dimer twofold. With one exception (WT:Glu versus K531A:Glu, at 50°), they were oriented at approximately 80° relative to the line joining the D1 centres of mass (table 3). As this approximates to the plane of the D1:D1 interface, these movements affect the distance between residues at the top and bottom of the interface. The shifts in K531A and K531A-T779G were such that residues at the top of the dimer were brought closer together, and those at the base left further apart. This movement was larger in the double mutant, suggesting that truncation of T779 to glycine allows closer approach of the protomers at the top of the interface.

**Table 3.** Domain movements in GluK2 apical mutants. n.a., not applicable; n.d., no rigid-body motion reported by dyndom.

GluK2 mutant	cleft-closure (Glu versus KA)	D1–D1 shift^a^ rotation (vector angle) (°)	
	rotation (°)	glu	KA
WT^b^	3.8 ± 0.6	1. 2.5 (44)	3. n.a.
		2. n.a.	
K531A^b^	6.4 ± 0.8	1. 4.7 (81)	3. 6.8 (83)
		2. 5.1 (49)	
K531A-T779G^b^	3.8 ± 0.8	1. 1.6 (64)	3. 6.6 (80)
		2. 7.1 (80)	
R775A	4.2 ± 0.5	1. 2.3 (49)	3 n.d.
		2. n.d.	

^a^The values represent the angle of rotation and the angle between the rotational vector and a line joining the centres of mass (in parentheses). Comparisons are for Glu complex versus KA complex (1), WT:Glu versus mutant Glu complex (2) and WT:KA versus mutant KA complex (3).

^b^Values for GluK2 WT are from Nayeem *et al.* [7]. Note that K531A:Glu and :KA complexes were determined in different space-groups, and the K531A-T779G CD protomer dimer was not included in comparisons because of incomplete ligand exchange.

There were also differences in the orientation of the D1:D1 interface depending on which ligand was bound. We had previously identified such ligand-dependent shifts when comparing GluK2 WT:Glu and :KA complexes [7]; these were matched in R775A, with an angular rotation of 2.3° in the D1:D1 interface (oriented at 50°). For K531A, there was a 4.7° difference in the D1:D1 interface between the Glu and KA complexes (axis oriented at 80°), but the rotation was in the opposite direction to that seen in WT and R775A. For K531A-T779G, in contrast, there was little difference (less than 2°) between the interface orientation of the Glu and KA complexes (table 3).

Overall, there were therefore significant differences in the conformation of the dimer interface in K531A-containing mutants compared with GluK2 WT. An alternative way to quantify these movements is by measuring the distances between residues at the top and bottom of the dimer. In GluK2 WT, the Cα atoms of R775 at the dimer apex are between 13.5 Å (Glu complex) and 13.2 Å apart (KA complex). This distance is reduced in both K531A (Glu complex 12.3 Å; KA complex 12.2 Å) and K531A-T779G (12.2 and 12.1 Å). Residues at the base of the dimer were correspondingly further apart in K531A and K531A-T779G. The usual measure taken is the Cα–Cα distance between either K544 or P667, as these residues flank the glycine–threonine linker that replaces the pore domain in the LBD construct. Movement here is therefore thought to reflect the transduction of conformational changes in the binding domain to the pore. For K544, differences compared with GluK2 WT ranged between 3.0 (K531A:Glu) and 4.2 Å (K531A:KA); for P667, they ranged from 2.2 (K531A-T779G:KA) to 3.1 Å (531A:KA). A more nuanced picture emerged when Glu and KA complexes were compared for each mutant. In GluK2 WT, the Cα–Cα distances are greater in Glu than KA (K544 by 0.5 Å; P667 by 1.2 Å), consistent with the notion that separation reflects efficacy. This was also the case for K531A-T779G (1.0 Å; 1.7 Å) and R775A (1.6 Å; 1.7 Å). The single exception was K531A, where the difference was −0.8 Å for K544 (and +0.5 Å for P667).

The changes in dimer conformation we observed are consistent with the attenuation of macroscopic desensitization in the K531A and K531A-T779G mutants. The larger interface shift in K531A:KA compared with :Glu also matched the observed linker separations (and the change in relative efficacy). To explore this further, we looked at binding-cleft closure, which is the usual proxy for efficacy in AMPA and kainate iGluRs. It is typically expressed as an angular movement of the D2 domain relative to a reference structure. Comparing cleft-closure with GluK2 WT:Glu (PDB code 2xxr), we found no rigid-body movements of D2 in either K531A-T779G:Glu or R775A:Glu. In K531A:Glu, in contrast, a 4.2° movement of D2 was required to match the WT:Glu conformation. Comparing the KA complexes, R775A:KA was again indistinguishable from WT:KA, but movement in D2 was seen in both K531A:KA (4.0°) and K531A-T779G:KA (4.1°).

Visual inspection showed these angular movements did not result from the usual ‘cleft-closure’ movement, however. We therefore determined the movement of the D2 domain along the three principal axes, based on the relative positions of the D2 centres of mass [7,35]. For this analysis, we used a GluK1 WT:Glu complex (PDB code 3fuz) as a reference structure [36], because a wider range of ligand complex structures, including antagonists, have been determined for the GluK1 LBD. The observed shifts confirmed that changes in ‘conventional’ cleft closure did not underlie the D2 domain movements in K531A and K531A-T779G. Closure of the LBD cleft in AMPA and kainate iGluRs is associated with movement of D2 along the first and third principal axes (figure 5*c*, left graph; equating to ‘up’ and ‘out’ motions as oriented). The various GluK2 Glu and KA structures were each clustered together, with the KA structures universally exhibiting more ‘open’ structures. The approximately 4° angular motions we observed in K531A and K531A-T779G compared with WT result instead from differences in the extent of movement along the second principal axis (i.e. sideways shifts in D2; figure 5*c*, right graph). In GluK2 WT and R775A (figure 5*c*,*d*), the D2 domains of the KA complexes are shifted ‘rightwards’ compared with the respective Glu complex. This is also the case for GluK1 structures in complex with partial agonists and antagonists (figure 5*c*). This shift is much smaller in K531A-T779G, underlying the 4.1° difference in D2 domain conformations in K531A-T779G:KA versus WT:KA. The real outlier, however, is K531A, for which the direction of this shift is reversed (figure 5*c*,*e*). Given the reversed efficacy seen in K531A, both this movement and the K544 Cα–Cα distance show a better correlation with efficacy than does cleft closure.

## 5. Discussion

Structure–function analyses of non-NMDA receptor desensitization focus largely on the stability of the LBD dimer. For the mutants described here, K531A, K531A-T779G and R775A, their structural effects divide into those with an obvious positive effect on dimer stability (i.e. changes to side chain and dimer conformation), and those without (i.e. reduced chloride binding). Truncation of K531 to alanine has clear stabilizing effects. The R775 and D776 side chains form two new inter-protomer interactions, namely cation-π bonding between R775 residues and a new contact between D776 and sodium. This is associated with movement in the dimer interface, bringing the apex closer together and base residues further apart. These changes closely match the effect of the D776K mutation, where the introduced lysines displace sodium and a similar anti-parallel interaction is formed by R775 side chains. In D776K, the combined effect of these changes is to bring protomers closer together at the dimer apex [7], increasing LBD dimer stability and blocking macroscopic desensitization. The attenuation of desensitization observed in the K531A and K531A-T779G mutants is therefore fully consistent with the structural changes we observed in the LBD dimers.

The observed lack of anion binding in the apical mutant structures is more problematic, both in terms of defining its potential effect on dimer stability, but also because exchanging chloride for iodide still affected K531A-T779G desensitization kinetics. In looking to explain the disparity between the structural and functional data, we think it highly unlikely that the LBD structures misrepresent the situation in the intact receptor. Ion binding sites depend on local geometry, and reduced anion binding would be expected in all these mutants, even if their phenotypes were not. There are therefore two possibilities. First, chloride ions may still bind to the interface and affect receptor function, but not be visible in electron density maps because of lower occupancy or a more poorly defined binding site (or both). A potentially analogous situation is observed in the GluK1 Cs^+^ complex described by Plested *et al*. [12], where a conformational change in R790 (homologue of GluK2 R775) opens up the anion site, resulting in more diffuse chloride binding. In our GluK2 mutants, the continued effect of chloride-exchange on desensitization in the absence of a strong amplitude effect might in this case reflect differing underlying ‘potencies’. The second, more speculative, possibility is that faster desensitization in NaI is the result of iodine binding to other sites in the receptor. To distinguish between these possibilities, it will be necessary to carry out a more detailed analysis of the functional effects of anions on these three mutants.

In terms of dimer stability, either of the possible explanations is still consistent with a role for reduced chloride binding. This is particularly relevant for R775A, where a lack of visible chloride binding is the only significant structural change. The association of reduced or absent chloride binding with slower KAR desensitization is surprising. Chloride binding has been shown to stabilize the GluK2 LBD dimer [29], and its loss should therefore promote desensitization. We observe the reverse effect, implying increased dimer stability. A possible explanation is that in the apical mutants the charge balance in this region (zero, counting 2× aspartate plus either 2x lysine or 2x arginine as appropriate) is actually more favourable to dimer stability than that in GluK2 WT (+1; 2× lysine, 2× arginine, 2× aspartate and 1× Cl^−^). This is still consistent with destabilization of GluK2 WT by removal of chloride, as the relative charge becomes +2. The presence of the anion may therefore serve to fine-tune dimer stability.

While the desensitization phenotypes of all three apical mutants can therefore be explained in terms of dimer stability, for a complete picture, we must consider other receptor states. In particular, it is the stability of transition states that determine kinetics. Mutations that attenuate desensitization are generally assumed to have stabilized the active state (or states). All things being equal, this decreases *ΔG*_des_ and increases *ΔG*^++^, resulting in a larger steady state (smaller *K*_eq_) and slower desensitization kinetics (larger *τ*_des_). Comparing changes in these parameters (figure 2*d*), there is a marked correlation for mutants described by Chaudhry *et al.* [10] located on the edge of the D1 domain (pink surface in figure 1*a*). For our apical mutants, a correlation is weaker but still evident. The slope is also steeper; in simple terms, the steady-state response is more sensitive to mutations in the dimer apex (figure 2*d*).

What can we conclude from this? The above discussion relies on two main mechanistic simplifications: that desensitization is a first-order process, and that the steady-state response represents re-entry into the peak open-state. The former is clearly not the case for our mutants (we plotted *τ*_1_ values for this reason), so we should not expect any link between *ΔG*_des_ and *ΔG*^++^ to be completely linear. On the source of the steady-state response, the existence of multiple open and desensitized states means the steady-state may result from transition into an open state other than from *O*_peak_. In this case, *ΔG*_des_ and *ΔG*^++^ would relate to different transitions. In the absence of a comprehensive gating model for GluK2, these comparisons are still worthwhile, however. While the observed correlations do not prove a causal link, they do indicate a connection between the stability of the open, desensitized and corresponding transition *states*. It is likely that the multiple states result at least in part from the tetrameric quaternary structure, and as such mutations are likely to affect all states in an equivalent (if not identical) way.

It is therefore likely that the apical and ‘D1-edge’ mutants both stabilize the LBD dimer, but with divergent effects on the stability of transition state(s) for exit from desensitization. Specifically, the apical mutants appear to stabilize these transition states as well as the active state. One possible source of this difference is the anion binding site, which is affected in all three mutants. If the dissociated dimer conformation identified by Armstrong *et al*. [3] represents the desensitized state, then transition from this to the LBD dimer we observe may well be affected by the electrostatics around the anion binding site. In other words, the fact that this region is (more) charge neutral in the apical mutants may lower the energy barrier to dimer dissociation.

In addition to affecting desensitization, the K531A mutation also affected ligand efficacy, but without affecting binding-cleft closure. Instead, we observed a correlation between sideways movement in the lower (D2) lobe and the relative efficacy of Glu and KA. In both GluK1 and GluK2 WT, this sideways shift is greater in partial than in full agonists, and greater still in three GluK1 LBD structures with antagonists bound (figure 5*d*). When ranked by this sideways shift, Glu bound to K531A groups with partial agonists, whereas KA groups with full agonists. We would predict from this that the efficacy of KA at K531A has risen, and that of Glu fallen, although single channel recordings would be needed to confirm this. Similar efficacy-related movements have been reported in GluA2 [37], so this may represent a general marker for ligand efficacy in AMPA and KARs. Of course, it should be stressed that LBD conformations either may be dependent on other domains in the receptor or may be constrained by crystal contacts. Furthermore, the weak partial-agonist dysiherbaine and its derivatives bind to an LBD conformation indistinguishable from full agonists [36,38] in terms of both cleft-closure and the extent of sideways shift in D2 (data not shown). Therefore, further elements, potentially outside the LBD, must be involved in determining efficacy.

Ultimately, any conformational changes in the LBD must be transmitted to the pore, and we also observed a correlation between efficacy and the Cα–Cα distance between K544 residues at the base of the dimer. Notably, the K544 Cα atoms are *further apart* in K531A:KA than in K531A:Glu, whereas in all other cases, separation was greater in the Glu complexes. As this distance reflects a combination of domain movements, including the angular conformation of the D1:D1 interface and the sideways shift in D2, it may prove a better marker for the effects of these mutants on receptor responses. This leads to a final related point. Despite its location away from the binding pocket, truncation of K531 affects the conformation of D2 relative to D1. This may result from changes at the D1:D1 interface, but might also be mediated through the hinge leading from D1 to D2 (i.e. F533 to L536). In either case, it raises the interesting possibility that ligands binding to this region of KARs (either interacting with or displacing the bound ions) could modulate both desensitization kinetics and ligand efficacy in a predictable way. Several AMPA-selective allosteric modulators bind to the base of the dimer interface, and our improved understanding of the role of these apical interactions may enable the development of kainate-selective equivalents.

## 6. Acknowledgements

We are grateful to Kate Davis for technical assistance with mutant generation and cell culture, Dr Yihong Zhang for initial electrophysiological characterization of the mutants, the beamline staff at the DIAMOND and BESSY-II synchrotrons and Steve Heinemann for the rat GluK2(Q) cDNA. Synchrotron beam-time at DIAMOND was made available through funding to the Manchester/Liverpool Block Allocation Group (BAG).
